# Equine pituitary pars intermedia dysfunction: Identifying research priorities for diagnosis, treatment and prognosis through a priority setting partnership

**DOI:** 10.1371/journal.pone.0244784

**Published:** 2021-01-04

**Authors:** Rebecca C. Tatum, Cathy M. McGowan, Rachel S. Dean, Joanne L. Ireland

**Affiliations:** 1 Institute of Ageing and Chronic Disease, University of Liverpool, Liverpool, United Kingdom; 2 Centre for Preventive Medicine, Animal Health Trust, Newmarket, Suffolk, United Kingdom; 3 Centre for Evidence-based Veterinary Medicine, University of Nottingham, Sutton Bonington, Leicestershire, United Kingdom; King Saud University, SAUDI ARABIA

## Abstract

Pituitary pars intermedia dysfunction (PPID) is the most prevalent endocrine disorder of older equids. To date, key research areas likely to have the greatest impact on equine health have not been identified. In human medicine, public and patient involvement is widely used to inform research agendas. This study aimed to engage with veterinary surgeons and horse owners to identify evidence gaps (‘uncertainties’) and prioritise these into a list of the 10 most important PPID research questions. The James Lind Alliance (JLA) Priority Setting Partnership (PSP) Framework was adapted. Questions about the diagnosis, treatment and prognosis of PPID were gathered via an online survey targeting veterinary surgeons and horse owners with experience of PPID. Thematic analysis was used to form a longlist of collated indicative research questions (CIRQs), defined by the JLA as true ‘evidence uncertainties’ when not answered by a published, clinically relevant, up-to-date systematic review. In an interim prioritisation survey, questions were ranked by weighted scores creating a shortlist of 25 that were taken forward to the PSP workshop, where participants reached a consensus on the top 10. Useable responses containing ≥1 question were received from 524 respondents (92.6% owners, n = 485; 7.4% veterinary surgeons, n = 39). After screening for relevance, 1,260 individual questions were included in thematic analysis, resulting in 47 CIRQs. Interim prioritisation votes for the CIRQs were received from 360 respondents. The top 10 questions prioritised at the PSP workshop focused on long-term prognosis, diagnostic accuracy, efficacy of pergolide treatment, alternative treatment/management strategies and potential treatment options for poor responders to pergolide. The quantity of questions generated indicates an extensive number of uncertainties regarding the diagnosis, treatment and prognosis of PPID. The top 10 research questions will help to inform key areas for evidence synthesis and knowledge translation, and to direct future research into areas most important to end users involved in caring for and treating animals with PPID.

## Introduction

Pituitary pars intermedia dysfunction (PPID) is a common age-associated equine neurodegenerative disorder [1,2]. While the exact pathophysiology of the disease remains poorly understood [3,4], oxidative stress is thought to contribute towards progressive neurodegeneration of the inhibitory dopaminergic hypothalamic neurons [1,5]. This leads to a loss of dopaminergic inhibition of the pars intermedia lobe of the pituitary gland and over production of pituitary-derived hormones. As a result, increased plasma concentrations of pro-opiomelanocortin (POMC) peptide and its derivatives, including α-melanocyte stimulating hormone, corticotropin-like intermediate lobe peptide, β-endorphin and adrenocorticotrophic hormone (ACTH), are observed [4,6,7]. This proliferation of hormones is associated with a variety of clinical signs and comorbidities including hypertrichosis, laminitis, epaxial muscle wastage or muscle atrophy and lethargy [2,8–10]. Current published evidence about diagnosing and managing PPID is limited, both in terms of the study populations included and the outcomes measured. Various different endocrinologic assay tests have been developed for the diagnosis of PPID. However, a ‘gold standard’ laboratory test is currently lacking [11,12]. Once diagnosed, the dopamine agonist pergolide is currently the only licensed treatment for PPID [13,14]. However, evidence regarding its efficacy is largely based on a single uncontrolled trial [14] and numerous descriptive reports [13,15–19]. Therefore, a more robust evidence base is required to inform veterinary surgeons and horse owners regarding optimal methods for diagnosis and medical treatment of PPID.

Ensuring that research is relevant and applicable to those who can improve patient care is essential. However, researchers do not always investigate areas or answer questions important to the end users [20,21]. Inclusive research methods in medicine have been developed to bridge the gap between patients, clinicians and researchers, encouraging widening of perspectives to identify gaps in the evidence and prioritise research agendas [22–24]. One of these methods is the framework developed by the James Lind Alliance (JLA) Priority Setting Partnership (PSP). The JLA has been established as a platform for an independent integrated approach to setting research agendas [25,26]. It brings together patients, carers and clinicians on a “level playing field” to identify and prioritise unanswered research questions, known as ‘uncertainties’ [26–30]. Despite successful public and patient involvement (PPI) being used in priority setting for human medical research for over a decade [24,31], it has only recently been adapted for use in a veterinary setting. An adaptation of the JLA framework has previously been used to set priorities for research into the treatment of chronic kidney disease in cats [32]. However, to date such priority setting has not been applied to equine research.

Veterinary surgeons and horse owners with experience of PPID are best placed to identify questions about the disease most in need of answering. Therefore, the aim of this study was to engage these end users in order to identify their top 10 research priorities for the diagnosis, treatment and prognosis of PPID.

## Materials and methods

### Adaptation of the James Lind Alliance (JLA) framework

This study did not include animal participants, therefore international, national, or institutional guidelines for humane animal treatment are not applicable. This project received institutional ethical approval from the University of Liverpool and Animal Health Trust. Following institutional ethical approval, the six steps of the JLA framework [25] were adapted to identify research priorities for PPID:

1. Identification of, and contact with, collaborators to form a steering group.
2. Development and dissemination of surveys to the target audience to gather questions they have regarding PPID.
3. Collation, categorisation and refining of the questions submitted by participants into a longlist, and formatting these collated questions in PICO (population, intervention, comparison, outcome) format where possible.
4. Searching the literature to identify if the questions are ‘uncertainties’. An uncertainty is defined by the JLA as “a question which cannot be answered by a relevant, up-to-date systematic review” [25].
5. If >30 uncertainties are identified, interim prioritisation is undertaken to form a short list of 25 questions taken forward to the PSP workshop.
6. The organisation of a PSP workshop.

### Establishing a priority setting partnership

A steering group was established to run and oversee the Priority Setting Partnership (PSP). The steering group consisted of veterinary surgeons, including specialists in evidence-based medicine and equine internal medicine, as well as horse owners with experience of PPID. A protocol detailing the specific objectives of the PSP was developed in accordance with the JLA guidelines [29,33] (S1 Appendix) and adapted for use in an equine veterinary setting. The target population was veterinary surgeons and horse owners with experience of PPID. Boehringer Ingelheim Animal Health UK Limited (BI) was identified as a collaborator, and their extensive database of relevant participants facilitated survey dissemination.

### Survey development and distribution

A survey was developed to collect questions from respondents based on the JLA guidelines using the freely available online survey tool, Kwiksurveys [34] (S2 Appendix). The survey gathered questions regarding the diagnosis, treatment and prognosis of PPID from veterinary surgeons and horse owners with experience of the disease. Open questions with free text boxes were used to facilitate this, and respondents could enter as many or as few questions as desired. An invitation to participate in the survey was distributed via email to collaborator BI’s “Care and Connect” database (a service which enables owners and veterinary surgeons to monitor horses with PPID after diagnosis) [35] and BI’s veterinary practice contact list: a tailored introduction was included explaining the purpose of the research. A link to the survey was also promoted through BI’s Talk About Laminitis Facebook page, and the respective websites and/or social media pages of the University of Liverpool Equine Hospital, University of Nottingham Centre for Evidence Based Veterinary Medicine (CEBVM) and Animal Health Trust. The aim was to reach as many relevant participants as possible. Enrolment efforts were targeted within Great Britain (GB); however, participation was not limited to respondents from GB. The survey was available online for eight weeks from 13^th^ April 2017 to 9^th^ June 2017.

### Processing the responses

**Fig 1:**
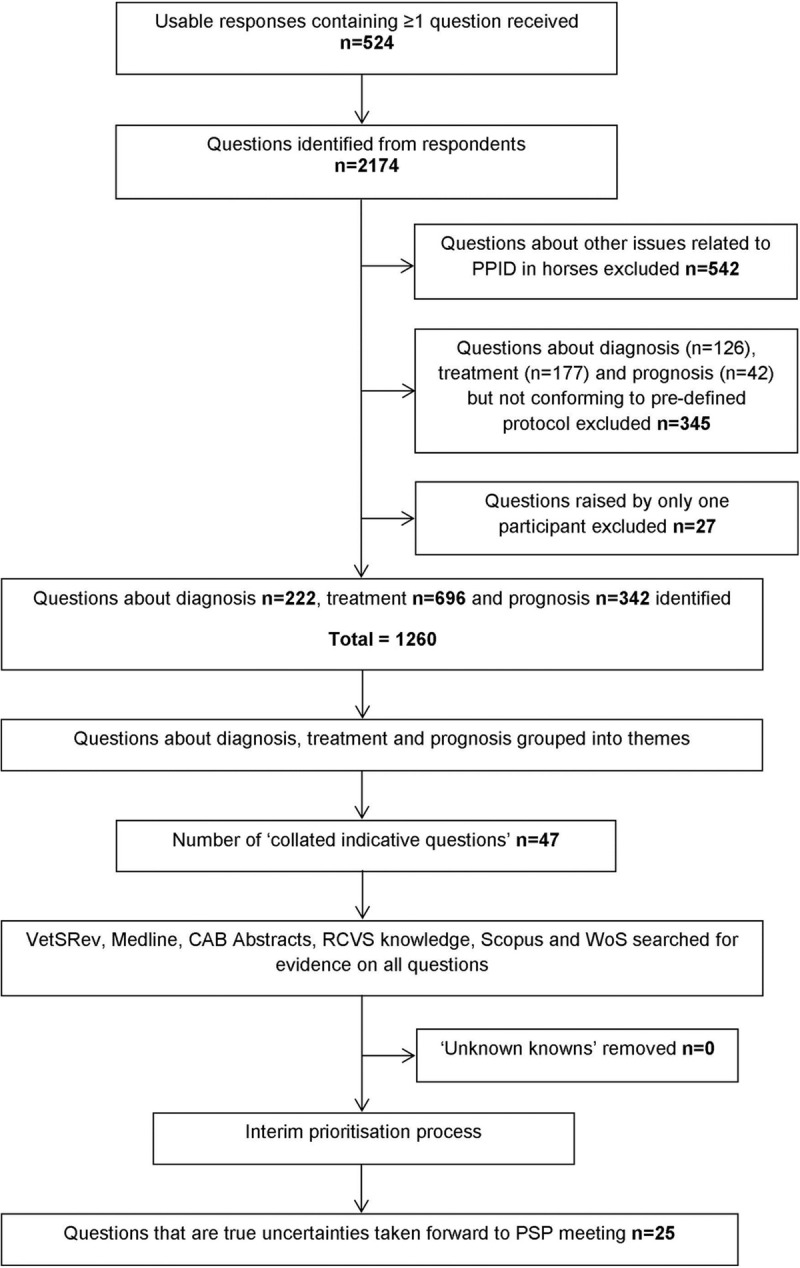
Process by which survey responses were converted to a shortlist of questions for prioritisation at the priority setting partnership workshop.

Responses were downloaded into a Microsoft Excel spreadsheet and anonymised. Each individual question was screened for relevance and to ensure the inclusion and exclusion detailed in the JLA PSP protocol (S1 Appendix) were met. Questions outside the specific objectives of the PSP were excluded. Questions about diagnosis, treatment and prognosis of PPID were systematically categorised into themes. Duplicates and similar submissions were then interpreted and combined to create indicative questions to enable searching of the evidence base (Fig 1). For example, questions about the various possible side-effects of pergolide treatment were combined to form the CIRQ ‘In horses with PPID, what are the side-effects of pergolide treatment both long and short-term?’. This enabled the themes and issues raised by the survey to be captured and made accessible to a non-research audience.

### Verification of uncertainties

The literature was searched for relevant, up-to-date systematic reviews using appropriate databases including MEDLINE, CABI, SCOPUS, Web of Science (WOS) and the online database of citations for systematic reviews of relevance to veterinary medicine and science VetSRev [36]. Broad search terms were used to ensure all relevant evidence was located (S1 Table). The literature regarding diagnosis, treatment and prognosis of PPID was identified and categorised into type of study: [i] systematic review, [ii] narrative review, [iii] clinical trial, [iv] case-control study, [iv] observational study [prospective/retrospective], [v] cross-sectional study, [vi] descriptive reports and [vii] “other” (which included all other types of study including conference abstracts or where study design could not be determined).

### Interim prioritisation

From the initial survey, more than 30 uncertainties were identified; therefore an interim survey was undertaken to prioritise which uncertainties should be included in the PSP workshop (S4 Appendix). A link to the interim survey was sent via email to all original survey respondents. To encourage additional responses, especially from veterinary surgeons, a link was also shared via the University of Liverpool Equine Practice Facebook page and other relevant social media pages replicating the original survey dissemination. This interim stage required participants to select their top 10 most important questions from the longlist, but not to rank them. The interim prioritisation survey was available online from 23^rd^ October 2017 to 20^th^ November 2017. Questions were ranked via weighted scores, depending on the number of votes received, to ensure equal weighting of veterinary surgeon and horse owner responses. Separate rankings were then combined to form a final ranked list of all questions. The top 25 questions were taken forward to final PSP workshop.

### Priority setting workshop

Participants from both surveys who expressed an interest in participating further, and had provided contact details for this purpose, were invited to attend the final PSP workshop. The final priority setting was conducted in a face-to-face workshop using both small and whole group discussions, utilising an adapted nominal group technique [29,37]. This allowed horse owners and veterinary surgeons to contribute equally to the discussion. The workshop was chaired and facilitated by the authors. To ensure transparency, each participant had to disclose who they were, what they did and any competing interests they had, for example if they worked in the pharmaceutical industry. Three rounds of prioritisation were undertaken using visual cue cards to rank questions; two small group sessions and one final whole group session. To ensure an even distribution of participants, veterinary surgeons and owners were assigned an identification number and randomly allocated to a group using a random number generator prior to the workshop [38]. The groups were purposively changed after the first session to ensure diverse collaboration and opinion sharing. After each round of prioritisation, the rankings from each small group were combined to form an overall aggregate rank for each question. The final ranking was then discussed and amended in the final whole group session with particular attention being paid to the top 10 collated questions.

## Results

### Respondent demographic information

A total of 524 usable responses, which contained at least one question about diagnosis, treatment and/or prognosis, were received from veterinary surgeons and/or horse owners with experience of PPID in the initial online survey. The majority of responses were from owners (92.6%; n = 485), of which 438 currently owned a horse with PPID and 47 had previously owned a horse with PPID. The remaining 39 responses were from veterinary surgeons, of which 35 were currently in practice treating horses with PPID, and four were also current owners of a horse with PPID. The majority of respondents were from GB (92.4%; n = 484), and 7.3% (n = 38) were from other regions including North America (n = 12), Europe (n = 10), Ireland, the British Channel Islands and Isle of Man (n = 9), Australasia (n = 6) and Asia (n = 1). A further two respondents did not provide information regarding their country of residence.

### Collation and rationalisation of questions

The process of rationalising the list of questions is summarised in Fig 1. The 524 participants submitted a total of 2,174 individual questions about PPID in horses/ponies. The responses were very variable: some contained one question and others contained multiple questions. Some respondents did not submit a question at all and simply described their experiences or asked about their own horse specifically. These were utilised where relevant statements were made but otherwise excluded. Each response was broken down into possible questions and themes for the next step of the process, in which 1,260 questions specific to diagnosis, treatment and prognosis that met pre-defined criteria (S1 Appendix) were identified. After thematic analysis, 47 CIRQs were identified.

Literature searches conducted on 06^th^ December 2017 (S1 Table) identified 134 relevant publications (after removal of duplicates), however no relevant up-to-date systematic reviews were identified (Table 1). Therefore, all 47 CIRQs from the survey were classed as unanswered, and consequently uncertainties, as defined by the JLA, and were taken forward to the two stage prioritisation process. After interim prioritisation, the 25 questions ranked highest overall were taken forward to the final PSP workshop (S2 Table). Examples of the questions submitted by respondents, how they were categorised to form the CIRQs and their ranking following interim prioritisation are shown in Table 2.

**Table 1:** Study design and main topics of relevant publications regarding pituitary pars intermedia dysfunction identified via a literature search conducted on 06/12/2017 [n = 134].

	Systematic Review	*Narrative Review	*Clinical Trial	*Case Control	*Observational studies [prospective/retrospective]	*Cross-sectional	*Descriptive reports	*Other
Diagnosis of PPID	0	12	0	20	9	3	26	14
Treatment of PPID	0	29	4	3	4	2	19	4
Prognosis of PPID	0	6	0	0	3	1	3	1

*Some studies covered multiple topics and therefore appear in the table more than once.

**Table 2:** The interim prioritisation rankings of the final top 10 questions agreed as shared priorities at the pituitary pars intermedia dysfunction priority setting partnership workshop, including examples of the original questions submitted and how they were categorised to form collated indicative research questions.

Number of respondent questions which contributed to the final collated question	Example questions*	Collated indicative research question	Interim veterinary surgeon rank	Interim owner rank	Interim overall rank [veterinary surgeon and owner rankings combined]
45	• What is the likely progression of the disease or is it very individual. • How will it develop and what are the signs that it is developing? • Is there any way of assessing likely rates of deterioration of PPID?	In horses with PPID, what is the expected disease progression over a horse’s lifetime both with and without treatment?	4	1	1
20	• Should the dosage vary at different times of year/when horse shows different symptoms? I realise there are seasonal variations in the ACTH levels. Can the Prescend [*sic*] be safely reduced during off peak times? • And should the amount of medicine change through the seasons?	In horses with PPID, does the dose of pergolide need to vary with the season?	3	9	2
13	• By treating with Prascend, and lowering ACTH levels to within the normal range, how much will the likelihood of symptoms reduce? E.g. reduce laminitis risk by 70%, skin infections by 60% • Are they still at the same risk of laminitis as they were before treatment was started? • Is it true that if you reduce the prascend that you may cause laminitis?	In horses with PPID receiving treatment with pergolide, is the risk of laminitis reduced?	2	10	3
75	• Is there anything else that can be done to slow this disease apart from prascend? • If Prascend stops working what other treatments are available? Can radiotherapy or surgery be used?	In horses with PPID, are there any medical treatments, other than pergolide, that work?	8	5	4
31	• Can results be inaccurate if a horse is stressed? • Can results be inaccurate if a horse is in pain? • Can other conditions such as ir and ems cause a false positive?	In horses with PPID, does stress, concurrent illness and/or pain affect the reliability and accuracy of diagnostic tests?	1	13	5
9	• Some cases refractory to treatment and difficult to explain why • What is the best way of treating apparent "non-responders" to pergolide? • What if treatment doesn't have the desired results?	What is the best way of dealing with horses who do not respond to pergolide treatment?	5	16	7
110	• What "lifestyle changes" should be made to help with the condition? • how can I keep my pony healthier while having PPID? • Are there any published studies of effective management and feed strategies for horses with PPID that can educate horse owners?	In horses with PPID, what additional management strategies [i.e. feed & turnout] are best to use in conjunction with pergolide treatment?	18	4	8
91	• Long term side effects of Prascend? • What are the side effects of the tablets? • Have you documented dysphagia as a side effect to prascend?	In horses with PPID, what are the side-effects of pergolide treatment [both long and short-term]?	17	7	9
3	• What is the recommended treatment/course of action after the horse has reached the recommended maximum daily number of Prascend tablets [i.e. 3 per day] and the ACTH levels are continuing to increase? • At what point in the progression of PPID is it advisable to discontinue increasing doses of pergolide? • What happens when you get to the maximum dose of prascend?	In horses with PPID, what should be done when the maximum dose of pergolide has been reached but hormone levels are still elevated?	16	12	11
67	• Homeopathic remedy that is as effective? • Have any herbal remedies ever been tested to work? • Are herbal/alternative treatments really effective in any way?	In horses with PPID, are any non-prescription treatments [i.e. Agnus Castus, homeopathy, other herbal products] effective?	37	2	16
11	• What is more important, clinical signs or bloods? What if the two don't match? • Should we diagnose an old horse without any clinical symptoms but a high ACTH in the blood, as having PPID? • What is the recommended plan for horses displaying symptoms of PPID but not testing positive?	In horses with suspected PPID, what is the best way to deal with inconclusive or conflicting test results and/or clinical signs [symptoms]?	10	35	21

*The raw questions are as they were entered by respondents, only spelling has been corrected.

### The priority setting partnership workshop

**Fig 2:**
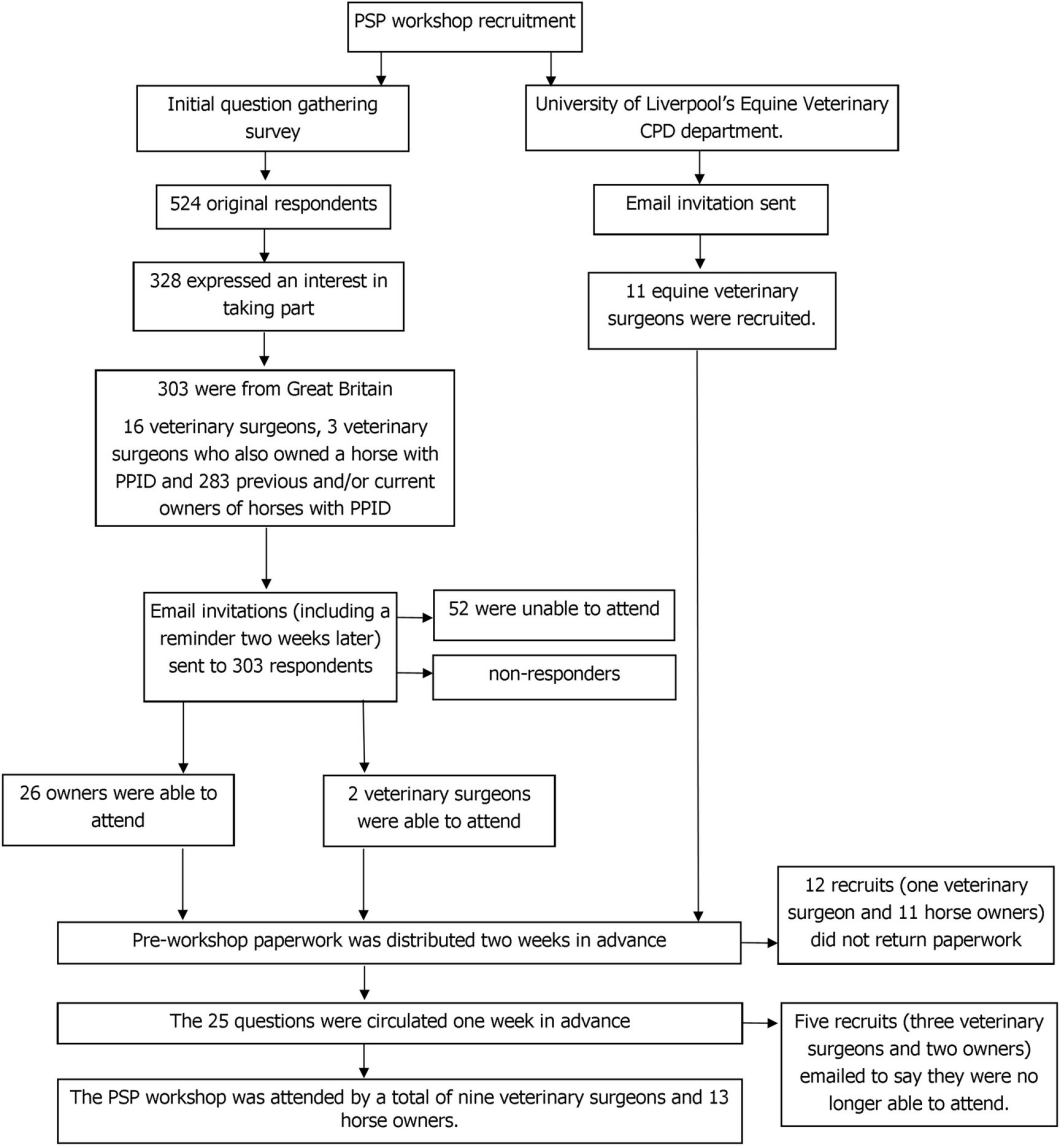
The process by which attendees were recruited to attend the PPID priority setting partnership workshop.

The PSP workshop was attended by a total of nine veterinary surgeons and 13 horse owners with experience of PPID (Fig 2). A list of the top 10 uncertainties for PPID was agreed by consensus.

During the small group discussion rounds, each group decided on their shared list of priorities. Following the second round of small group discussions, there was little change overall in the order of the top eight questions. However, there was some change in the middle rankings. During the third whole group discussion, the final shared prioritised list of questions was agreed (Table 3). Overall, there was general consensus on the ranking for the majority of questions with the exception of question P (‘In horses with PPID, are any non-prescription treatments (i.e. Agnus Castus, homeopathy, other herbal products) effective?’). There was much debate around this question and owners put forward a case for it to be moved into the top 10. However, agreement could not be reached on which question should be moved out of the top 10 in order to accommodate question P. Throughout the workshop discussions, participants considered that question P was closely related with question H (‘In horses with PPID, what additional management strategies (i.e. feed & turnout) are best to use in conjunction with pergolide treatment?’). Therefore, it was decided that the two questions could be encompassed under a single ranking and that question P would form a ‘part b’ to question H (ranked fourth).

**Table 3:** Final shared top ten research priorities for the diagnosis, treatment and prognosis of pituitary pars intermedia dysfunction (PPID) ranked during the PPID priority setting partnership workshop.

ID	Question	Final Order
A	In horses with PPID, what is the expected disease progression over a horse’s lifetime both with and without treatment?	1
E	In horses with PPID, does stress, concurrent illness and/or pain affect the reliability and accuracy of diagnostic tests?	2
D	In horses with PPID, are there any medical treatments, other than pergolide, that work?	3
H	In horses with PPID, what additional management strategies [i.e. feed & turnout] are best to use in conjunction with pergolide treatment?	4a
P	In horses with PPID, are any non-prescription treatments [i.e. Agnus Castus, homeopathy, other herbal products] effective?	4b
C	In horses with PPID receiving treatment with pergolide, is the risk of laminitis reduced?	5
I	In horses with PPID, what are the side-effects of pergolide treatment [both long and short-term]?	6
B	In horses with PPID, does the dose of pergolide need to vary with the season?	7
U	In horses with suspected PPID, what is the best way to deal with inconclusive or conflicting test results and/or clinical signs [symptoms]?	8
G	What is the best way of dealing with horses who do not respond to pergolide treatment?	9
K	In horses with PPID, what should be done when the maximum dose of pergolide has been reached but hormone levels are still elevated?	10

Following the PSP workshop, participants were sent a feedback questionnaire. The feedback questionnaire was completed by 16 attendees (five veterinary surgeons and eight owners, three did not specify). Median participant ratings for their overall experience, usefulness, purpose and organisation of the PSP workshop out of 10, were 9.5, 9, 10 and 10, respectively.

## Discussion

This PSP achieved engagement with horse owners and veterinary surgeons across two phases of prioritisation to identify research priorities for PPID. The JLA framework was successfully applied to an equine disease of complex pathophysiology, indicating this methodology could be effectively applied to other equine diseases. The research priorities, within the pre-defined topics of diagnosis, treatment and prognosis, included: [i] long-term prognosis, [ii] diagnostic accuracy, [iii] efficacy of pergolide, [iv] alternative treatment/management strategies and [v] potential treatment options for poor responders to treatment with pergolide. As the study was conducted in GB and respondents were primarily from GB, differences in horse populations, management and treatment practices around the world means the questions raised here may not be globally representative.

The PSP followed a rigorous and transparent predefined process to create a top 10 list of shared research priorities. The process gathered a large number of responses, comparable with other JLA PSPs [39,40]. The JLA does not define a target sample size. Instead, a saturation point is reached where no new themes are emerging [29]. Participants posed multiple questions resulting in numerous themes and a large but manageable number of questions. At the point the survey was closed, no new themes were emerging.

Various methods were used to distribute the survey with some being more successful than others. Advertising via social media was effective for recruiting horse owners but not veterinary surgeons. A large number of veterinary surgeons were contacted via BI’s mailing list. However, the number of veterinary responses compared to horse owners was disappointing. This may have been for a number of reasons, such as they were not interested in engaging in this type of research, the open question design of the questionnaire discouraged them from participating or they felt there were no gaps in the evidence and therefore did not have a question to pose. A more targeted approach or a prior, formal collaboration with veterinary practices may have improved veterinary responses. However, a low response rate from veterinary surgeons and veterinary practices has been noted in other studies [41,42]. Exploring motivators and barriers for veterinary involvement in research may improve future engagement.

Despite the low number of responses, questions posed by participating veterinary surgeons did not markedly differ from those posed by owners. Around a quarter of questions raised by participants were not specific to diagnosis, treatment or prognosis and instead covered other PPID-related subjects. This is comparable the previous veterinary PSP conducted by Dean *et al* (2014) [32]. The majority of these non-useable questions were regarding the disease’s pathophysiology which remains poorly understood [8]. Many of the questions collated were un-structured and non-specific. Therefore, in order to form structured questions which enabled searching for evidence, questions were adapted and combined to form CIRQs. This stage was a qualitative process, with a set structure and technique set out by the JLA followed to ensure consistency [33]. This allowed questions to be combined and managed without losing context. A limitation of the process is that some of the research questions identified may represent a failure of communication, knowledge transfer or understanding, rather than actual evidence gaps. For example, submitted questions such as ‘what diagnostic tools are available?’ and ‘what is it you are actually testing the levels of in the blood sample?’ indicate that horse owners may not fully comprehend the diagnostic process. Therefore, the validity of each CIRQ was carefully considered by the steering group to ensure they were unanswered research questions, as defined by the JLA.

The ‘Choose Ten’ interim prioritisation approach was chosen because it is an uncomplicated way of allowing participants to consider the whole list then make choices that involve genuine shortlisting [29]. Despite additional promotion, the number of responses from veterinary surgeons for the second interim survey remained low. The scoring and ranking method ensured that votes from veterinary surgeons and owners were equally weighted. A difference in priorities between groups was observed; some questions ranked highly by veterinary surgeons were ranked considerably lower by horse owners and vice versa. For example; ‘In horses with PPID, does stress, concurrent illness and/or pain affect the reliability and accuracy of diagnostic tests?’ was ranked highest by veterinary surgeons but thirteenth by owners.

During the adaptation of the JLA protocol, it was decided that the JLA definition of an ‘uncertainty’ would be applied for the equine veterinary setting. There are few systematic reviews in equine medicine; at the time of writing only 23 were available on the VetSRev database. This meant it was likely all questions posed by participants would be defined as uncertainties. However, it was agreed by the steering group that the level of certainty could not be lowered and the quality of evidence should remain the same across fields. Systematic reviews collate data from numerous studies and offer the highest level of evidence [43], and if the JLA definition was changed it may have mislead PSP participants regarding the level of evidence available [32]. The evidence base found for the diagnosis, treatment and prognosis of PPID was of poor to moderate quality [43]. While no published systematic reviews pertaining to PPID were available prior to this PSP, equine endocrinology has been identified as a fast‐moving field [44] and a large number of studies published within the past two decades contribute some evidence towards the top 10 research questions reported here. Future knowledge synthesis, such as systematic reviews, focused on these questions could offer an opportunity to close some of these evidence gaps. Importantly, this PSP has also identified a requirement to raise awareness within the equine veterinary profession of the need for better dissemination of the findings from previous research.

The final PSP workshop allowed an open and thoughtful exchange of views between horse owners and veterinary surgeons. This enabled consensus to be developed and facilitated the identification of the top 10 research questions. Both groups were represented by an appropriate number of participants [29,33]. All groups worked well together, with veterinary surgeons raising issues relating to owners and vice versa. As in other PSP workshops, changes in ranking after group discussions were noted [45]. For example, the question ‘In horses with PPID, how effective is pergolide at slowing the progression of the disease?’ was ranked seventh and third by veterinary surgeons and owners respectively during the interim prioritisation, but was not prioritised into the final top 10. This is evidence of good group discussion and decision making, suggesting the ability to overcome biases [45]. The number one prioritised question, ‘In horses with PPID, what is the expected disease progression over a horse’s lifetime both with and without treatment?’ was ranked highly throughout the prioritisation process. Pituitary pars intermedia dysfunction is a chronic progressive disease associated with several co-morbidities [1,2,4,46]; it is therefore unsurprising this question was ranked highest. It is a broad question encompassing several elements and therefore in the process of answering this question, it is possible a number of the other top 10 questions may also be answered. Several studies have investigated the initial efficacy of pergolide [13,14,47–50]. However, there is very little evidence regarding long-term effectiveness of treatment for improving prognosis. One small study investigated treatment response after 5.5 years and found owners of surviving horses were satisfied with clinical response [51], and a recent retrospective study reported increased odds of short-term survival (median 11 months) in PPID cases treated with pergolide [52].

Two of the top 10 questions related to the accuracy and interpretation of diagnostic tests. In several studies, utilising a variety of different clinical reference standards, the commonly used basal ACTH test has been reported to have good sensitivity and specificity [11,50,53–55]. However, uncertainty remains regarding test accuracy for PPID diagnosis in the presence of factors that may affect ACTH levels (Table 3, question E), such as concurrent disease (particularly insulin dysregulation), stress [56] and pain [57]. In referral hospital populations, a number of acute conditions have been reported to result in elevations of ACTH [57,58]. To date, these pre-analytical factors have not been evaluated in a population of PPID cases. However, the high proportion of systemically ill horses with ACTH concentrations above the upper limit of the reference interval at hospital admission [58] indicates that it is important to consider these factors when interpreting basal plasma ACTH concentration for the diagnosis of PPID in practice. The second question encompassed respondents’ uncertainly regarding the relative importance of endocrine laboratory test results and observed clinical signs. This included results that do not ‘fit the clinical picture‘, such animals exhibiting clinical signs of PPID but with normal ACTH concentrations, as well as the interpretation of equivocal or ‘borderline’ test results and the best way to manage these situations (Table 2).

The majority of questions centred around treatment (Table 3), with questions focusing on additional management strategies, effective medical treatments other than pergolide [D], dosing of pergolide throughout the year [B], safety [I] and efficacy of pergolide treatment [C, G and K]. Several studies have reported that pergolide is effective at improving clinical signs and ACTH levels [14,19,51], and this was generally the view point of participating veterinary surgeons at the beginning of the PSP. However, discussions with owners throughout the process highlighted that this is not always the case for individual animals. The process highlighted the need for a more robust evidence base for pergolide as a treatment and the need to investigate concurrent and alternative options.

Priority questions identified in this PSP are potentially methodologically complex to answer in terms of study design, implementation, ethical concerns and financial limitations. The JLA process is not concerned with how the questions raised will be answered: its function is to provide a platform for the involvement of end users [25–27]. However, the breadth of each topic offers researchers the opportunity to develop future studies dependant on resources available. Although all questions are considered important, it may not be possible to fund or answer all of them. In addition to supporting the direction of future research, the top 10 questions identify specific issues that horse owners consider important within each topic, providing a valuable resource to inform targeted owner education.

This study shows that horse owners and veterinary surgeons can be involved in identifying and prioritising uncertainties. The involvement of veterinary surgeons and owners at this stage of the research process has the ability to improve available evidence, ensure research is relevant to end users and aid decision making. During the PSP process horse owners acted as a proxy for the patient. The JLA has previously been utilised in this way for feline medicine [32]. This adaptation is comparable to other JLA PSPs where patients cannot speak for themselves and carers or parents represent them, for example those involving children [59]. The best interest of the patient remains the focus in each case.

## Conclusion

The JLA methodology can be successfully adapted into an equine veterinary setting and applied to the diagnosis, treatment and prognosis of PPID in horses. The response and quantity of questions generated indicates an extensive number of uncertainties about the disease. However, as the research was undertaken in GB, it is possible that the research questions prioritised may differ from unanswered questions of veterinary surgeons and horse owners in other countries. Identifying the top 10 research questions for a disease or condition, especially those that require long-term management, will help to direct evidence synthesis, knowledge translation and future research into areas most important to the end users.

## Supporting information

S1 Table Details of search terms used and databases searched. (PDF)

S2 Table The questions ranked highest after interim prioritisation and taken forward to the priority setting partnership workshop. (PDF)

S1 Appendix James Lind Alliance priority setting partnership protocol for identifying research priorities for diagnosis, treatment and prognosis of pituitary pars intermedia dysfunction. (PDF)

S2 Appendix James Lind Alliance uncertainty gathering survey. (PDF)

S3 Appendix Interim prioritisation survey. (PDF)

S4 Appendix Included original diagnosis questions submitted to the uncertainty gathering survey. (XLSX)

S5 Appendix Included original treatment questions submitted to the uncertainty gathering survey. (XLSX)

S6 Appendix Included original prognosis questions submitted to the uncertainty gathering survey. (XLSX)
