# Supplementary material for: Equine pituitary pars intermedia dysfunction: Identifying research priorities for diagnosis, treatment and prognosis through a priority setting partnership
Source: PLoS One. 2021 Jan 4;16(1):e0244784. doi: 10.1371/journal.pone.0244784 (PMC7781667; doi:10.1371/journal.pone.0244784)
Supplement: S1 Table — (PDF) [file pone.0244784.s001.pdf]

S3 Appendix

Details of search terms used and databases searched when evaluating evidence for the diagnosis, treatment and prognosis of pituitary pars intermedia dysfunction

Databases searched	Medical Literature Analysis and Retrieval System Online (MEDLINE) 1966-2017<br><br>Centre for Agriculture and Bioscience International (CABI) 1972-2017<br><br>Elsevier's abstract and citation database (SCOPUS) 1966-2017<br><br>Web of Science (WOS) 1945-2017	Via University of Liverpool library
	VetSRev	Via Centre for Evidence-Based Veterinary Medicine
Search terms:	Horse* OR pony OR ponies OR equine* OR equid* AND Pituitary pars intermedia dysfunction OR PPID OR cushing* OR pituitary neoplasia OR hyperadrenocorticism OR pituitary adenoma*	
Dates searches performed:	06/12/2017	
