# Supplementary material for: Equine pituitary pars intermedia dysfunction: Identifying research priorities for diagnosis, treatment and prognosis through a priority setting partnership
Source: PLoS One. 2021 Jan 4;16(1):e0244784. doi: 10.1371/journal.pone.0244784 (PMC7781667; doi:10.1371/journal.pone.0244784)
Supplement: S2 Table — (PDF) [file pone.0244784.s002.pdf]

S4 Appendix

The 25 questions ranked highest overall after interim prioritisation and taken forward to the priority setting partnership workshop

Question ID	Question
A	In horses with PPID, what is the expected disease progression over a horse's lifetime both with and without treatment?
B	In horses with PPID, does the dose of pergolide (Prascend) need to vary with the season?
C	In horses with PPID receiving treatment with pergolide (Prascend), is the risk of laminitis reduced?
D	In horses with PPID, are there any medical treatments, other than pergolide (Prascend), that work?
E	In horses with PPID, does stress, concurrent illness and/or pain affect the reliability and accuracy of diagnostic tests?
F	In horses with PPID, how effective is pergolide (Prascend) at slowing the progression of the disease?
G	What is the best way of dealing with horses who do not respond to pergolide (Prascend) treatment?
H	In horses with PPID, what additional management strategies (i.e. feed & turnout) are best to use in conjunction with pergolide (Prascend) treatment?
I	In horses with PPID, what are the side effects of pergolide (Prascend) treatment (both long and short term)?
J	In horses with PPID, what is the best method of monitoring response to treatment so that dose alterations can be made?
K	In horses with PPID, what should be done when the maximum dose of pergolide (Prascend) has been reached but hormone levels are still elevated?
L	What is the prognosis for horses with PPID?
M	In horses with PPID, what is the best way to manage asymptomatic cases (a horse diagnosed with PPID but with no symptoms)?
N	In horses with PPID, do co-existing illnesses (such as equine metabolic syndrome, arthritis or laminitis) affect prognosis?
O	In horses with PPID, what is the best way to manage side effects of pergolide (Prascend) treatment?
P	In horses with PPID, are any non-prescription treatments (i.e. Agnus Castus, homeopathy, other herbal products) effective?
Q	In horses with PPID, how do we improve prognosis?
R	Could alternative preparations of pergolide (Prascend) be effective (i.e. liquid preparation or injection) when treating PPID in horses?
S	In horses with PPID, what is the best way to manage borderline cases?
T	In horses with PPID, how effective is pergolide (Prascend) treatment at reducing/controlling clinical signs (symptoms) of PPID?
U	In horses with suspected PPID, what is the best way to deal with inconclusive or conflicting test results and/or clinical signs (symptoms)?
V	In horses with PPID, do factors such as diet, management or time of day affect the reliability and accuracy of diagnostic tests?
W	In horses with PPID, what is the most cost effective way of monitoring the disease?
X	In horses with PPID, how long is pergolide (Prascend) treatment effective for?
Y	In horses with PPID, what are the long term effects of the disease?
