# Supplementary material for: Equine pituitary pars intermedia dysfunction: Identifying research priorities for diagnosis, treatment and prognosis through a priority setting partnership
Source: PLoS One. 2021 Jan 4;16(1):e0244784. doi: 10.1371/journal.pone.0244784 (PMC7781667; doi:10.1371/journal.pone.0244784)
Supplement: S1 Appendix — (PDF) [file pone.0244784.s003.pdf]

S1 Appendix

Pituitary Pars Intermedia Dysfunction Priority Setting Partnership

PROTOCOL 15/06/2017

Adapted from the James Lind Alliance protocol

Purpose of the PSP and background

The purpose of this protocol is to set out the aims, objectives and commitments of the Pituitary Pars Intermedia Dysfunction (PPID) Priority Setting Partnership (PSP) and the basic roles and responsibilities of the partners therein.

The Pituitary Pars Intermedia Dysfunction PSP will be led and managed by the primary investigator PhD student Becky Tatum BSc (Hons) (BT).

PSP lead and advisors (Steering Group)

Advisors to the student investigator will comprise of equine veterinary surgeons Dr Jo Ireland (JI) and Prof Cathy McGowan (CM) of the University of Liverpool, as well as horse owning members of the Animal Health Trust Epidemiology department, two of whom are also veterinary surgeons. Methodological guidance will be provided by veterinary surgeon and Director of the Centre of Evidence-based Veterinary Medcine (CEVM) Dr Rachel Dean (RD). These representatives of horse owners and veterinary surgeons form the steering group for this PSP.

Background to the Pituitary Pars Intermedia Dysfunction Priority Setting Partnership

PPID is the most prevalent endocrine disorder in older horses and is therefore of significant importance in equine practice. It is a progressive endocrine disease which causes laminitis, abnormal coat changes and fat distribution among other clinical signs. The available evidence regarding the diagnosis, treatment and prognosis of PPID is limited both in terms of the study populations included and the outcomes measured. An evidence base is needed to inform veterinary surgeons and owners, yet the quality of published evidence is fair to poor. Therefore, it is important that any research done in this area is applicable to practice and can be used directly by those that can improve patient care. The evidence gaps in the diagnosis, treatment and prognosis of PPID need to be identified and prioritised.

Aims and objectives of the Pituitary Pars Intermedia Dysfunction PSP

The aim of the PPID PSP is to identify the unanswered questions (known as uncertainties) about PPID diagnosis, treatment and prognosis from horse owner and clinical perspectives, then prioritise those that horse owners and veterinary surgeons agree are the most important. A secondary aim is to ascertain if the JLA PSP framework can be applied to equine veterinary practice.

The objectives of the PPID PSP are to:

- Work with horse owners and veterinary surgeons to identify uncertainties about the accuracy, reliability and effectiveness of diagnosis techniques, including screening for PPID.
- Work with horse owners and veterinary surgeons to identify uncertainties about the efficacy and effectiveness of PPID treatments, including monitoring and response to treatment.
- Work with horse owners and veterinary surgeons to identify uncertainties about the prognosis of horses and ponies with PPID both long and short term including quality of life and influencing factors.
- To agree by consensus a prioritised list of those uncertainties, for research purposes
- To publicise the results of the PSP and process
- To take the results to research commissioning bodies to be considered for funding.

Partners

Organisations and individuals will be invited to be involved with the PSP as partners. Partners are groups or individuals who will commit to supporting the PSP by disseminating the PSP survey. Partners represent the following groups:

- Horse owners with experience of PPID
- Veterinary surgeons with clinical experience of PPID

Horse owners will be included in the PSP if they currently treat or care for at least one horse/pony with PPID or have done so in the past. Veterinary surgeons will be included in the study if they have clinical experience of diagnosing and/or treating PPID in horses/ponies.

It is important that organisations which can reach and advocate for these groups should be invited to become involved in the PSP. Boehringer Ingelheim Vetmedica Ltd (BI) has been engaged to collaborate with the project as they have contacts for both horse owners and veterinary surgeons with experience of PPID. To ensure wider coverage the Universities of Liverpool and Nottingham, Animal Health Trust were also engaged to collaborate.

Some organisations may be judged as having conflicts of interest. These can be perceived to adversely affect those organisations' views, causing unacceptable bias. This is likely to affect the ultimate findings of the PSP. As a pharmaceutical company BI is considered to have a conflict of interest. However, the Steering Group considers their partnership important to target a large number of relevant participants; therefore BI will participate in a purely observational capacity once the survey has been disseminated.

Methods

This section describes a schedule of proposed stages through which the PSP aims to fulfil its objectives. The process is iterative and dependent on the active participation and contribution of different groups. The

methods adopted at any stage will be agreed through consultation between the steering group members, guided by the PSP's aims and objectives. More details can be found in the Guidebook section of the JLA website at www.jla.nihr.ac.uk where examples of the work of other JLA PSPs can also be seen.

Stage 1: Identification and invitation of potential partners and raising stakeholder awareness

Potential partner organisations will be identified through a process of peer knowledge and consultation, through the Steering Group members' networks. Potential partners will be contacted and informed of the establishment and aims of the PPID PSP.

Stage 2: Identifying uncertainties

An online survey will be developed to identify questions veterinary surgeons and horse owners have about PPID. The survey will be based on the guidelines outlined in the JLA guidebook and adapted through consultation with the steering group. BI, the Animal Health Trust, Veteran Horse Society and the Universities of Liverpool and Nottingham will identify a method for distributing the initial survey to potential participants, to enable questions and uncertainties of practical clinical importance relating to the diagnosis, treatment and prognosis of PPID to be identified. A period of 6 - 8 weeks will be given to complete this exercise or until no new themes are emerging.

Refining questions and uncertainties

The consultation process (survey) will produce "raw" unanswered questions about diagnosis, treatments and prognosis. These raw questions will be assembled and categorised and refined by BT supervised by RD into "collated indicative questions" which are clear, addressable by research and understandable to all.

Questions will undergo two initial rounds of refinement, firstly to remove questions not relating to diagnosis, treatment or prognosis. Such as prevalence, prevention, risk factors, pathophysiology and associated diseases.

Secondly out-of-scope submissions about diagnosis, treatment and prognosis which do not fall under the specific aims and objectives of the study will be removed. This will include, for example, questions relating to understanding diagnostic test results, availability of treatment options/how PPID is treated or whether there is a 'cure' for the disease.

Relevant statements which can be formatted or merged into questions will be included where appropriate.

Relevant submissions will be further refined by removing:

- Questions submitted by only one participant
- Incomplete submissions which do not define the respondents characteristics i.e. whether they are a veterinary surgeon or horse owner with experience of PPID (this information is needed to ensure the transparency of the refining process)
- Submissions from respondents that do not have experience of PPID

- Submissions from respondents with experience of PPID in donkeys or non-domestic equines

Categorisation and thematic analysis will be used to identify similar or duplicate questions which will be combined where appropriate. Questions will then be reformatted and reworded where necessary to form research questions understandable to all.

Existing sources of information about uncertainties for owners and clinicians will be searched. This will include research recommendations in systematic reviews and other evidence; clinical trials, narrative reviews and clinical guidelines. To identify sources of uncertainties and research recommendations MEDLINE, CABI, SCOPUS, WOS and VetSRev databases will be searched. The existing literature will be researched to see to what extent these refined questions have, or have not, been answered by previous research.

If questions are expressed can be answered with reference to existing research evidence (systematic reviews that meet the JLA criteria of certainty) and are therefore "unknown knowns" and not uncertainties, this evidence will be highlighted by the PSP to their membership. This suggests that information is not being communicated effectively to those who need it. A separate record of these 'answerable questions' will be kept.

Uncertainties that are not adequately addressed by previous research will be collated for interim (if applicable) and final prioritisation. The checking undertaken to demonstrate that the uncertainties have not already been answered will be recorded.

Stage 3: Prioritisation – interim and final stages

The aim of the final stage of the priority setting process is to prioritise through consensus, the identified uncertainties relating to the diagnosis, treatment or prognosis of PPID. This will be carried out by members of the Steering Group and the wider partnership that represents horse owners and veterinary surgeons.

The interim stage

The refining process will result in a longlist of indicative uncertainties, the number of which is hard to predict. If the longlist of indicative uncertainties is >30 it will be reduced to a shortlist to be taken forward and discussed at the final PSP workshop. This will be achieved through an interim prioritisation survey circulated to participants of the initial survey and via relevant social media. Participants in this round of prioritisation will be invited to choose the 10 uncertainties most important to them from the longlist. They will not be asked to rank them. The responses obtained will be used to rank the uncertainties by number of votes. The top 25 will be taken forward into the final PSP workshop.

The final stage

The process to reach a shared list of 10 most important uncertainties will be conducted in a face-to-face workshop, using group discussions and plenary sessions. This will involve participants from the previous stages that represent horse owners and veterinary surgeons. The method used for this prioritisation process will be an adapted nominal group technique overseen by members of the steering group to ensure equal contribution from participants.

The aim is to hold one workshop including questions about diagnosis, treatment and prognosis. However, if the number of questions regarding these individual areas is deemed by the Steering Group to warrant three separate PSP workshops, treatment uncertainties will be prioritised in the face-to-face workshop while uncertainties about diagnosis and prognosis will be prioritised online using adapted Delphi technique.

Findings, research and publicity

Findings and research

It is anticipated that the findings of the PPID PSP will be reported to the pharmaceutical industry and major research funding charities as well as other funding bodies for clinical veterinary research . Relevant Steering Group members and partners will publish the 'top 10' priorities on their websites and in various publications to horse owners and the veterinary profession. All partners will be encouraged to develop the prioritised uncertainties into research questions, and to work to establish the research needs of those unanswered questions to use when approaching potential funders, or when allocating funding for research themselves, if applicable.

As well as alerting funders, partners and advisory group members the results will be published using both internal and external communication mechanisms. This will be done in an open access format that is easy to understand to all participants and potentially interested funders of research. The findings will also be the topic of a peer reviewed paper and PhD thesis chapter. The production of an academic paper should not take precedence over publicising of the final results.
