# Supplementary material for: Equine pituitary pars intermedia dysfunction: Identifying research priorities for diagnosis, treatment and prognosis through a priority setting partnership
Source: PLoS One. 2021 Jan 4;16(1):e0244784. doi: 10.1371/journal.pone.0244784 (PMC7781667; doi:10.1371/journal.pone.0244784)
Supplement: S2 Appendix — (PDF) [file pone.0244784.s004.pdf]

S2 Appendix

Prioritising PPID – we need you

Welcome to the study investigating research priorities for Pituitary Pars Intermedia Dysfunction diagnosis, treatment and prognosis

Pituitary Pars Intermedia Dysfunction (PPID), also known as Equine Cushing's Syndrome, is a common hormonal disorder in older horses and ponies. It causes laminitis, abnormal coat changes and fat redistribution among other clinical signs (symptoms). There are still many unanswered questions regarding accurate diagnosis, effective treatment and the outcome or prognosis of PPID.

The purpose of this research is to engage with you, the vets and owners who treat and care for horses and ponies with PPID, in order to identify unanswered questions known as 'uncertainties'. Uncertainties are essentially unanswered questions that cannot be answered by up to date information based on research evidence. This survey is designed so that you can tell us the questions or issues you have about the diagnosis, treatment and prognosis of PPID. Establishing what you consider to be the most important questions in this area will help research organisations prioritise the studies that they fund. Ultimately this will mean that research projects are funded which aim to answer these questions, and overall improve the welfare of horses and ponies with PPID.

Identifying uncertainties is a fairly new concept in equine veterinary medicine. Examples of unanswered question in other areas might be:

• Which is the most reliable way to diagnose asthma?

• What is the best way of preventing fleas in dogs?

• What is the life expectancy of a cat with liver disease?

For further information, definitions and who to contact if you require assistance please Click Here

This survey is anonymous and any personal information provided will be kept confidential. The survey should take approximately 10 minutes to complete. **Please only complete this survey if you have experience of PPID** (for example, as a practising vet or by caring for a horse/pony with PPID).

This survey has been designed by Becky Tatum at the Animal Health Trust along with the Universities of Liverpool and Nottingham.

1* Please tick the box to indicate that you have read and understood the information about this study

○ Yes

Next Page

Your questions about PPID

2 What questions do you have about the diagnosis of PPID in horses/ponies? (Please write as many or as few questions as you like)

3 What questions do you have about the treatment of PPID in horses/ponies? (Please write as many or as few questions as you like)

4 What questions do you have about the prognosis (outcome) of horses/ponies with PPID? (Please write as many or as few questions as you like)

Previous Page Next Page

Treating PPID

If you do not currently own or care for a horse/pony diagnosed with PPID please continue to question 8 on the next page

5 If you currently own or care for a horse/pony with PPID, how long ago were they diagnosed?

- ○ Within the last 12 months
- ○ 1-3 years ago
- ○ Over 3 years ago

6 Did your horse/pony receive any medical treatment within the first 12 months after their diagnosis?

- ○ Yes, received Pergolide
- ○ No, did not receive treatment
- ○ Yes, Other (Please Specify)

7 If your horse/pony was diagnosed more than 12 months ago, are they still receiving medical treatment?

- ○ Yes, currently receiving Pergolide
- ○ No, not currently receiving treatment
- ○ Other (Please Specify)

Previous Page | Next Page

About you

8* Please select the option which best describes you

- ○ I am a veterinary surgeon who treats horses and ponies with PPID
- ○ I am an owner/carer who currently cares for a horse or pony with PPID
- ○ I am an owner/carer who has previously cared for a horse or pony with PPID
- ○ Other (Please Specify)

9 How did you find out about this study?

- ☐ Via Care and Connect
- ☐ Via the Animal Health Trust
- ☐ Via the Veteran Horse Society
- ☐ Directly from my veterinary surgeon
- ☐ Through a friend/colleague/client
- ☐ Via other social media

Other (Please Specify)

10 Where do you currently live?

- ○ Within Great Britain
- ○ Outside Great Britain (please specify where)

11* As part of this research we may publish the uncertainty/question that you, along with other participants, have identified. When reporting the results, no participants will be identified and your participation in this study will remain entirely anonymous. Please indicate if we have your permission to do so

- ○ Yes
- ○ No

Previous Page | Next Page

The next stage of the study

Once responses from owners and veterinary surgeons have been collected, volunteers will be needed to help us prioritise which of the questions identified are the most important. This will be done at a meeting in either the Newmarket or Liverpool area (depending on which is most convenient). The meeting will involve both owners and veterinary surgeons with experience of PPID who have participated in this survey. You do not need experience of participating in a meeting like this before to take part, and all contributions will be highly valued. Unfortunately limited funding means we are unable to pay participants for attending this meeting, however refreshments will be provided (for participating veterinary surgeons the meeting will count as CPD).

12 If you are interested in being involved in the next stage of this study please complete your contact details below. All personal information will be treated confidentially

Name

Address 1

Address 2

Town/City

County

Postcode

Contact phone number

Email address

13 If you would like to be contacted with the results of the study please provide your email address below

Previous Page | Finish Survey

Thank you for completing this survey.

Your contribution to our research is appreciated.

If you would like any more information please contact Becky Tatum at the Animal Health Trust: becky.tatum@aht.org.uk
