# Supplementary material for: Equine pituitary pars intermedia dysfunction: Identifying research priorities for diagnosis, treatment and prognosis through a priority setting partnership
Source: PLoS One. 2021 Jan 4;16(1):e0244784. doi: 10.1371/journal.pone.0244784 (PMC7781667; doi:10.1371/journal.pone.0244784)
Supplement: S3 Appendix — (PDF) [file pone.0244784.s005.pdf]

PPID - Identifying the top research questions

This study is investigating research priorities for Pituitary Pars Intermedia Dysfunction (PPID), also known as Equine Cushings Syndrome. PPID is the most common hormonal disorder in older horses and ponies in the UK. Despite this there are still has many uncertainties about the best ways to identify and manage this important disease.

This survey contains a long list of questions derived from over 2000 original submission made by vets and owners to our Prioritising PPID survey. We want to prioritise the top 10 questions from this list of 51 questions.

The order in which the questions appear will be different for each person completing the survey so that the questions which appear top of the list don't get more attention than the bottom.
Please identify the 10 questions that matter to you the most.

We will take the questions with the most votes from this survey forward to a final workshop. At this workshop we will bring together owners of horses with PPID and the vets that treat them to look at these questions and identify the most important ones based on the results of the survey. This will result in a 'top 10' list of research questions which will be used to help direct future research.

This survey is aimed at horse owners and veterinary surgeons with experience of PPID. **Please only complete this questionnaire if you have experience of PPID**, for example as an owner or carer of a horse with PPID or as an attending vet.

All information provided is anonymous and confidential. This survey was designed by Becky Tatum in collaboration with the Universities of Liverpool and Nottingham and Animal Health Trust for further information please **click here**.

1* **Please indicated that you have read the information provided and are happy for your survey response to be used for the purposes of this study (all information provided is anonymous and confidential)**

A Yes

Choosing your top 10

Please click on the **10** questions most important to you in the list below. You do not need to rank them.

2*

What are the ten questions you would like answering by future PPID research?

In horses with PPID what is the best time of day to give Pergolide (Prascend)?	In horses with suspected PPID when should secondary diagnostic tests be used?	Could alternative preparations of Pergolide (Prascend) be effective (i.e. liquid preparation or injection) when treating PPID in horses?
In horses with PPID what are the side effects of Pergolide (Prascend) treatment (both long and short term)?	In horses with PPID what additional management strategies (i.e. feed & turnout) are best to use in conjunction with medical treatment?	In horses with suspected PPID could non-invasive diagnostic tests be used to diagnose PPID?
In horses with PPID what is the best way to manage side effects of Pergolide (Prascend) treatment?	In horses with PPID how effective is Pergolide (Prascend) treatment at reducing/controlling clinical signs of PPID?	In horses with PPID do factors such as age, breed and diet influence the effectiveness of treatment?
In horses with PPID does stress affect the reliability and accuracy of diagnostic tests?	In horses with PPID what is the best way of dosing accurately with Pergolide (Prascend) tablets?	In horses with PPID does the dose need to vary with the season?
In horses with PPID what is the best method of monitoring response to treatment so that dose alterations can be made?	In horses with suspected PPID what are the clinical signs (symptoms) that should arouse suspicion of disease and therefore prompt a blood test to be done?	In horses with PPID treated with Pergolide (Prascend) how much dose variation is to be expected throughout an individual horse's treatment?
What is the best way of diagnosing PPID early?	In horses with PPID is it best to give Pergolide (Prascend) once or twice daily?	In horses with PPID what is the best treatment option?
Is routine screening of horses at risk of developing PPID beneficial?	In horses with PPID what is the best way to manage with asymptomatic cases (a horse diagnosed with PPID but with no symptoms)?	In horses with PPID are any non-prescription treatments (i.e. Agnus Castus, homeopathy or herbal remedies) effective?
What effect does the storage and handling of blood samples have on diagnostic test results?	In horses with suspected PPID what is the best time of year to perform diagnostic tests?	In horses with suspected PPID could other diagnostic tests aid in the diagnosis of PPID (for example measuring cortisol or different hormones)?
In horses with PPID in what time frame would you expect to see a response to treatment?	In horses with suspected PPID what is the most reliable and accurate primary diagnostic test to use?	In horses with PPID does the severity/stage of disease influence the effectiveness of treatment?
How often should horses at risk of developing PPID be screened?	In horses with PPID what should be done when the maximum dose has been reached but hormone levels are still elevated?	In horses with PPID what is the most cost effective way of monitoring the disease?

What is the prognosis for horses with PPID? Compared to horses without the disease?	In horses with PPID does concurrent illness and/or pain affect the reliability and accuracy of diagnostic tests?	In horses with PPID does treatment affect the prognosis?
In horses with PPID what is the expected disease progression over the horse's lifetime both with and without treatment?	In horses with PPID starting Pergolide (Prascend) treatment what is the best way to avoid initial side effects?	In horses with suspected PPID can a definitive diagnosis be made on clinical signs (symptoms) alone?
In horses with PPID receiving treatment with Pergolide (Prascend) is the risk of laminitis reduced?	In horses with PPID are there consequences if Pergolide (Prascend) doses are missed, if so what are they?	In horses with PPID what is the best way to manage borderline cases?
In horses with PPID are there any other medical treatments that work?	What is the best way of dealing with horses who do not respond to Pergolide (Prascend) treatment?	In horses with PPID do factors such as diet, management or time of day affect the reliability and accuracy of diagnostic tests?
In horses with suspected PPID what is the best way to deal with inconclusive or conflicting test results/clinical signs?	In horses with PPID how do we improve prognosis?	In horses with PPID how effective is Pergolide (Prascend) at slowing the progression of the disease?
In horses with PPID how long is Pergolide (Prascend) treatment effective for?	In horses with suspected PPID is a single diagnostic test sufficient to diagnose PPID, or is re-testing required to provide a conclusive diagnosis?	In horses with PPID do factors such as age, breed or management affect prognosis?
In horses with PPID what are the long term effects of the disease?	In horses with PPID does concurrent illness affect prognosis?	In horses with PPID does the stage/severity at diagnosis affect prognosis?

About you

3* **Did you take part in the initial Prioritising PPID survey?**

A Yes
B No
C Don't Know

4* **Which of the following best describes you?**

A I am a veterinary surgeon who treats horses and ponies with PPID
B I am an owner/carer who currently cares for a horse or pony with PPID
C I am an owner/carer who previously cared for a horse or pony with PPID
D Both a veterinary surgeon and owner/carer of a horse or pony with PPID
E Both a previous and current owner/carer of a horse or pony with PPID

Other (Please Specify)

5 **How did you find out about this study?**

A Via the Talk about laminitis (TAL) scheme
B Via the Animal Health Trust
C Directly from your veterinary surgeon
D Through a friend/colleague/client
E Via social media
F Via the University of Liverpool

Other (Please Specify)

6 **Where do you currently live?**

A Within Great Britain
B Outside Great Britain

If you currently live outside Great Britain please specify where

Previous Page | Next Page

The final stage of the study

The final stage of the study involves a workshop where vets and owners will come together to prioritise the questions with the most votes from this survey into a list of the 'top 10' most important research questions which need answering. The resulting top 10 will help direct future PPID research into the areas most important and useful to you, the end users. The workshop will be held in the Liverpool area on 8th December, you do not need experience of this kind of meeting to attend and all contributions are highly valued. Travel expenses will be reimbursed and for vets it counts as CPD.

7

If you would like to take part in the prioritisation workshop please complete you contact details below. All personal information will be treated confidentially

Name

Address

Address

Town

County

Postcode

Email Address

Phone Number

8

If you would like to be informed of the results of this study please provide your email address below

Previous Page | Finish Survey

Thank you for completing this survey and contributing towards our research.

If you would like any further information please contact Becky Tatum at becky.tatum@aht.org.uk

CREATE YOUR OWN FORM
